# In vivo characterization of a podocyte-expressed short podocin isoform

**DOI:** 10.1186/s12882-023-03420-x

**Published:** 2023-12-19

**Authors:** Linus Butt, David Unnersjö-Jess, Dervla Reilly, Robert Hahnfeldt, Markus M. Rinschen, Katarzyna Bozek, Bernhard Schermer, Thomas Benzing, Martin Höhne

**Affiliations:** 1 Department II of Internal Medicine, University Hospital Cologne CECAD building Joseph-Stelzmann-Str. 62, Cologne, 50931 Germany; 2 Center for Molecular Medicine Cologne (CMMC), Faculty of Medicine, University of Cologne, University Hospital Cologne, CECAD Building, Joseph-Stelzmann-Str. 62, 50931 Cologne, Germany; 3 Cologne Excellence Cluster On Cellular Stress Responses in Aging-Associated Diseases (CECAD), Faculty of Medicine, University of Cologne, University Hospital Cologne, Cologne, Germany; 4 MedTechLabs, Karolinska University Hospital, Solna, Sweden; 5 Department of Biomedicine and Aarhus Institute of Advanced Studies (AIAS), Aarhus University, Aarhus, Denmark; 6 Department of Medicine III, University Medical Center Hamburg-Eppendorf, Hamburg, Germany

**Keywords:** Podocin, Podocin isoform, Transgenic mouse, Slit-diaphragm morphology

## Abstract

The most common genetic causes of steroid-resistant nephrotic syndrome (SRNS) are mutations in the *NPHS2* gene, which encodes the cholesterol-binding, lipid-raft associated protein podocin. Mass spectrometry and cDNA sequencing revealed the existence of a second shorter isoform in the human kidney in addition to the well-studied canonical full-length protein. Distinct subcellular localization of the shorter isoform that lacks part of the conserved PHB domain suggested a physiological role. Here, we analyzed whether this protein can substitute for the canonical full-length protein. The short isoform of podocin is not found in other organisms except humans. We therefore analysed a mouse line expressing the equivalent podocin isoform (*podocin*^*Δexon5*^) by CRISPR/Cas-mediated genome editing. We characterized the phenotype of these mice expressing podocin^Δexon5^ and used targeted mass spectrometry and qPCR to compare protein and mRNA levels of podocin^wildtype^ and podocin^Δexon5^. After immunolabeling slit diaphragm components, STED microscopy was applied to visualize alterations of the podocytes’ foot process morphology.

Mice homozygous for *podocin*^*Δexon5*^ were born heavily albuminuric and did not survive past the first 24 h after birth. Targeted mass spectrometry revealed massively decreased protein levels of podocin^Δexon5^, whereas mRNA abundance was not different from the canonical form of podocin. STED microscopy revealed the complete absence of podocin at the podocytes’ slit diaphragm and severe morphological alterations of podocyte foot processes. Mice heterozygous for *podocin*^*Δexon5*^ were phenotypically and morphologically unaffected despite decreased podocin and nephrin protein levels.

The murine equivalent to the human short isoform of podocin cannot stabilize the lipid-protein complex at the podocyte slit diaphragm. Reduction of podocin levels at the site of the slit diaphragm complex has a detrimental effect on podocyte function and morphology. It is associated with decreased protein abundance of nephrin, the central component of the filtration-slit forming slit diaphragm protein complex.

**Supplementary Information:**

The online version contains supplementary material available at 10.1186/s12882-023-03420-x.

## Background

**Fig. 1:**
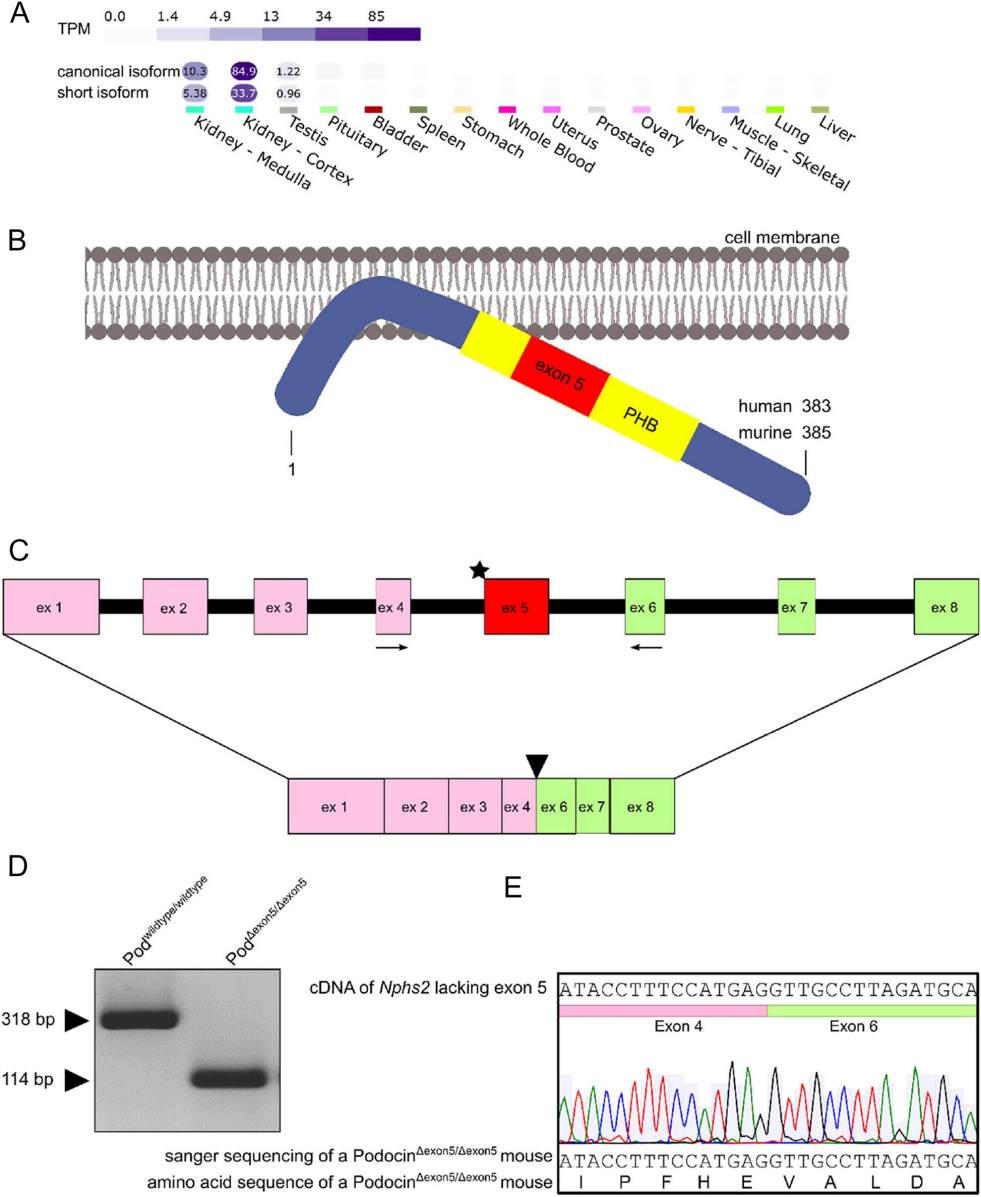
Podocin^Δexon5^ mice lack exon 5 due to a deletion affecting the splice acceptor site. **A** Podocin isoform mRNA expression across different tissues in healthy human individuals. TPM = transcripts per million. Data Source: GTEx Analysis Release V8 (dbGaP Accession phs000424.v8.p2). **B** Schematic illustration of the podocin protein. Exon 5 is located within the characteristic PHB domain. **C** The CRISPR/Cas9 mediated deletion in podocin^Δexon5^ mice abolishes the splice acceptor site of exon 5 leading to an exon skipping in the final mRNA. ★ = site of deletion,  = forward primer,  = reverse primer. Arrows indicate primers used for figure d. See Supplemental Fig. 1 for an amino acid alignment. **D** RT-PCR using the primers indicated in panel c. In podocin^Δexon5/Δexon5^ mice a piece of DNA representing exon 5 is missing. **E** Sanger sequencing of the cDNA of a podocin.^Δexon5/Δexon5^ mouse shows the in-frame transition from exon 4 directly to exon 6

*NPHS2*, the gene encoding podocin, is ranked among the most frequently mutated genes in patients with genetic steroid-resistant nephrotic syndrome (SRNS) [1] or focal and segmental glomerulosclerosis (FSGS) [2, 3]. Podocin is a hairpin-like membrane-associated protein that multimerizes to recruit plasma membrane lipids [4, 5]. The protein is expressed explicitly in podocytes, which are part of the glomerular filtration barrier. Based on human genetic and knock-out mouse studies, a central role of podocin for the development and maintenance of the glomerular filtration barrier and the physiological foot process morphology was demonstrated [6, 7]. Years of extensive research have shed light on its mutational spectrum [8–11] as well as on its role as a scaffolding and signaling protein at the slit diaphragm [4, 11–13]. The vast majority of human and mouse studies have focused on the canonical form of podocin, which is highly conserved between the two species (86% sequence identity and 95% sequence similarity) [14]. We have previously identified protein expression of a short isoform of podocin in the human kidney [15]. Interestingly, this isoform can only be found in humans. Available RNA sequencing data from the Genotype-Tissue Expression (GTEx) project revealed a surprisingly high expression level for the short isoform of about half the level of the canonical isoform in human kidney tissue (Fig. 1a). In the short isoform, exon 5 is lacking without affecting the reading frame of the remaining mRNA. The genomic region spanned by exon 5 is known to be enriched for the occurrence of mutations [16]. Experiments in cell culture could show that this isoform is mostly retained in the endoplasmic reticulum, which is a pathogenic feature known from other disease causing mutations of podocin [9, 17, 18]. In this study, we characterized a mouse line expressing a podocin^Δexon5^ allele resembling the human short isoform of podocin, which is physiologically not present in the mouse. Specifically, we addressed the open questions what the role of the short isoform is in vivo and whether implications for a rescue capacity of the short isoform can be found. The latter is of particular interest in cases in which disease-causing mutations are present in exon 5, which is missing in the short isoform.

## Results

### Podocin^Δexon5/Δexon5^ mice have a severe congenital phenotype

In the process of inserting a specific point mutation into exon 5 of podocin, a 255 bp deletion affecting the acceptor splice site of exon 5 occurred (Fig. 1b, see methods section “Genotyping” for specific information on the deletion) due to a non-homologous end-joining repair event during CRISPR/Cas9 mediated genome editing. As a result, exon 5 was skipped entirely from the resulting mRNA (Fig. 1c—e, Suppl. Figure 1). Importantly, this did not affect the reading frame of the remaining *Nphs2* mRNA as exon 5 lies in frame of the regular reading frame of the gene. Exon 5 encodes for the identical part of the protein in human and mouse, hence, the artificial murine short-isoform (Δexon 5) faithfully copies the human short-isoform (Suppl. Figure 1).

**Fig. 2:**
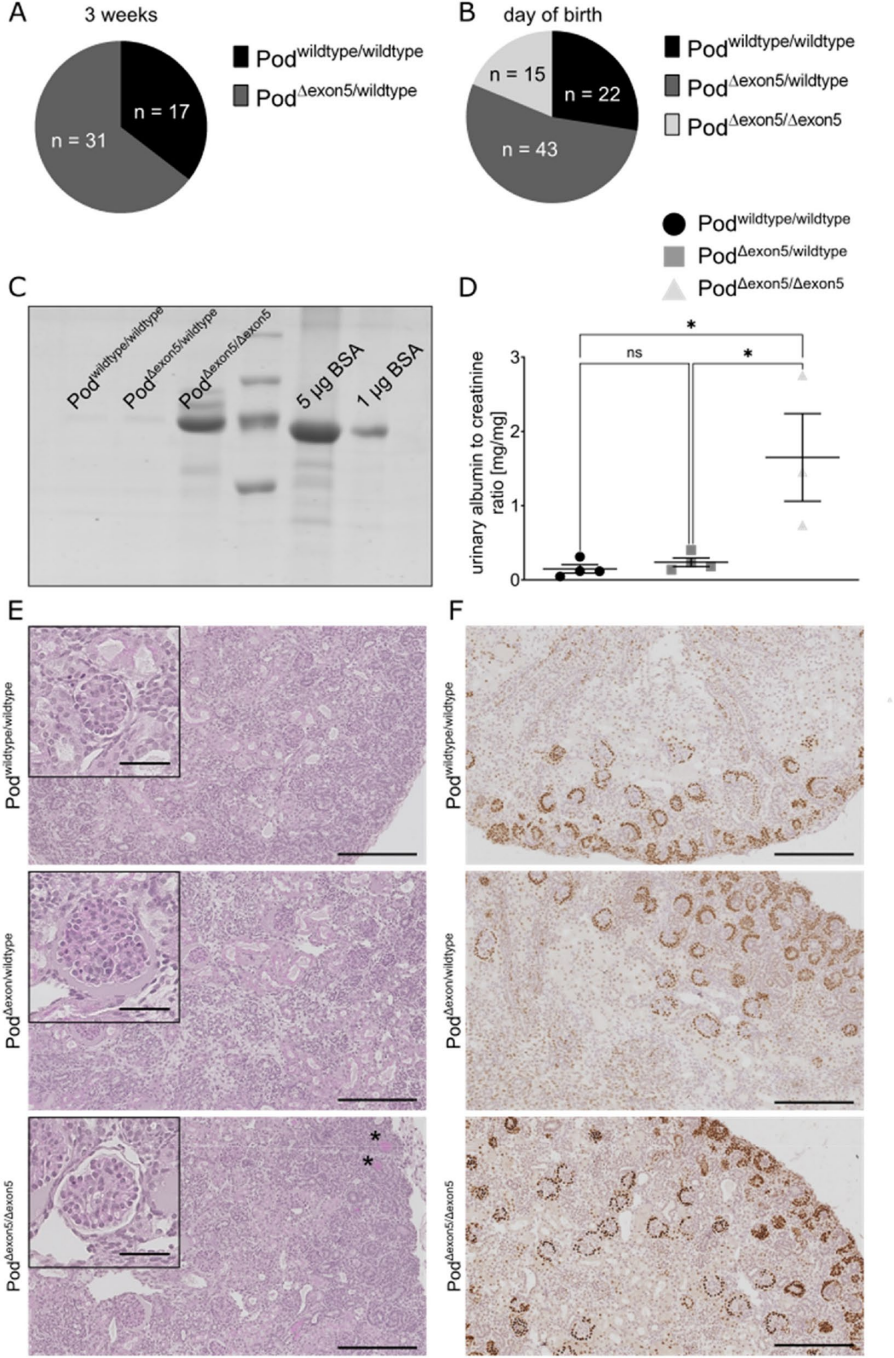
Podocin^Δexon5/Δexon5^ mice display a severe congenital phenotype. **A** Not a single podocin^Δexon5/Δexon5^ mouse could be observed at the age of weaning (~ 3 weeks, *n* = 48 mice). **B** At birth (P0) the distribution of genotypes is as expected, according to Mendelian laws (*n* = 80 mice). The expected ratio of observed genotypes was confirmed using a Chi-Square test. **C** Colloidal coomassie staining of urine samples from podocin^wildtype/wildtype^, podocin^Δexon5/wildtype^ and podocin^Δexon5/Δexon5^ mice at P0. As compared to the other two genotypes, more albumin could be detected in urine of podocin^Δexon5/Δexon5^ mice. Full-length gel is presented in Suppl. Figure 4. **D** Quantifications of the urinary albumin creatinine ratio of podocin^wildtype/wildtype^, podocin^Δexon5/wildtype^ and podocin^Δexon5/Δexon5^ mice at P0. The ratio was significantly higher in podocin^Δexon5/Δexon5^ mice. Tukey’s multiple comparison test was used to determine statistical significance. Data are presented as mean ± SEM (n ≥ 3 mice per genotype). * *p* < 0.05. **E** PAS staining of podocin^wildtype/wildtype^, podocin^Δexon5/wildtype^ and podocin^Δexon5/Δexon5^ mice at P0. No sclerotic lesions could be found, however multiple proteins casts were detectable in the tubular system of Podocin^Δexon5/Δexon5^ mice (asterisk). Scale bars correspond to 200 µm and 40 µm (insets), respectively. **F** Wt-1 Immunohistochemistry of podocin^wildtype/wildtype^, podocin^Δexon5/wildtype^ and podocin^Δexon5/Δexon5^ mice at P0. Distribution of Wt-1 positive cells was not globally altered among the three genotypes. Scale bars correspond to 200 µm

While heterozygous podocin^Δexon5/wildtype^ mice were phenotypically indistinguishable from wild-type mice, podocin^Δexon5/Δexon5^ mice were not detected at the age of weaning (~ 3 weeks, Fig. 2a). To exclude prenatal death, we investigated the genotypes on the day of birth (P0).

Genotyping a total number of 80 newborn pups revealed a regular distribution of genotypes according to Mendelian laws (Fig. 2b). It can therefore be concluded that podocin^Δexon5/Δexon5^ pups died between their birth and the age of weaning, which is similar to podocin knock-out mice [4]. 

Homozygous podocin^Δexon5/Δexon5^ pups showed a drastically increased albuminuria at the time of birth whereas there was no significant difference between podocin^wildtype/wildtype^ and podocin^Δexon5/wildtype^ mice (Fig. 2c and d). Strikingly, collecting urine samples from podocin^Δexon5/Δexon5^ mice was only possible in a subset of animals due to anuria in many pups while this problem was not present in podocin^wildtype/wildtype^ and podocin^Δexon5/wildtype^ mice. Histologically, no FSGS lesions could be found in podocin^Δexon5/Δexon5^ mice, yet protein casts could be observed in the tubular system of podocin^Δexon5/Δexon5^ mice (Fig. 2e, asterisk). Staining for Wt-1, a key transcription factor in glomerular development and a specific marker for developed podocytes, did not reveal substantial differences between the three genotypes (Fig. 2f) which indicates that the generation and differentiation of podocytes is not disrupted.

## Podocin and nephrin protein levels are decreased in podocin^Δexon5/wildtype^ and podocin^Δexon5/Δexon5^ mice

**Fig. 3:**
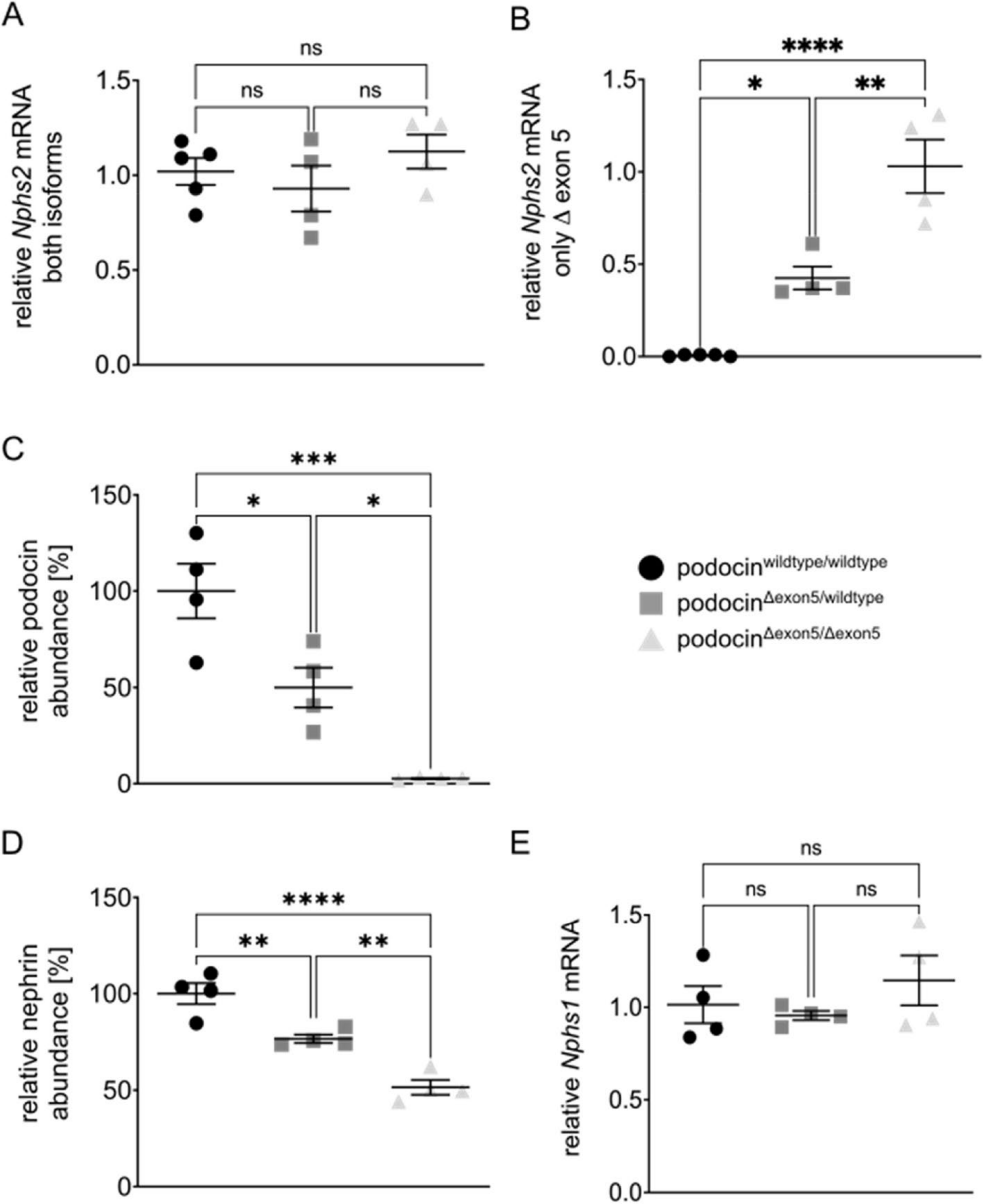
Quantifications of *Nphs1 and Nphs2* mRNA and podocin and nephrin protein in kidneys of podocin^wildtype/wildtype^, podocin^Δexon5/wildtype^ and podocin^Δexon5/Δexon5^ mice at P0. **A** Overall *Nphs2* mRNA levels were not significantly different among the three genotypes. **B** A custom designed qPCR assay specifically quantifies the *Nphs2*^Δexon5^ mRNA. **C** Using a targeted mass spectrometry approach, podocin abundance was measured in podocin^wildtype/wildtype^, podocin^Δexon5/wildtype^ and podocin^Δexon5/Δexon5^ mice at P0. Podocin levels were significantly lower in podocin^Δexon5/wildtype^ and podocin^Δexon5/Δexon5^ mice. **D** Using a targeted mass spectrometry approach, nephrin abundance was measured in podocin^wildtype/wildtype^, podocin^Δexon5/wildtype^ and podocin^Δexon5/Δexon5^ mice at P0. Nephrin levels were significantly lower in podocin^Δexon5/wildtype^ and podocin^Δexon5/Δexon5^ mice. **E** Quantification of *Nphs1* mRNA in kidneys of podocin^wildtype/wildtype^, podocin^Δexon5/wildtype^ and podocin.^Δexon5/Δexon5^ mice at P0. Tukey’s multiple comparison tests were used to determine statistical significance. Data are presented as mean ± SEM (*n* = 4 mice per genotype, each circle/square/triangle represents one mouse). * *p* < 0.05, ** *p* < 0.01, *** *p* < 0.001

Next, we assessed mRNA levels of the two distinct *Nphs2* isoforms. We used qPCR assays that allowed the mRNA quantification of both, *Nphs2*^wildtype^ and *Nphs2*^Δexon5^ (referred to as unspecific qPCR assay, Fig. 3a) or *Nphs2*^Δexon5^ only (Fig. 3b). There was no statistically significant difference between the three genotypes in levels of total *Nphs2* mRNA using the unspecific podocin qPCR assay (Fig. 3a). Using *Nphs2*^Δexon5^ isoform specific primers, we verified that *Nphs2*^Δexon5^ mRNA could not be detected in podocin^wildtype/wildtype^ mice and *Nphs2*^Δexon5^ mRNA levels were roughly 50% in podocin^Δexon5/wildtype^ as compared to podocin^Δexon5/Δexon5^ mice (Fig. 3b). Both findings emphasize the specificity of our qPCR approach. We then quantified protein abundances of podocin and nephrin, its binding partner and central protein of the slit diaphragm, encoded by the *Nphs1* gene. To this end, we set up a targeted mass spectrometry assay, which enabled us to specifically quantify podocin and nephrin peptides from kidney samples. Importantly, only podocin peptides outside of exon 5 were included in the assay in order to enable detection of podocin^Δexon5^ protein. Contrary to the similar total podocin mRNA levels between the three genotypes, podocin protein levels were significantly different among all genotypes (Fig. 3c). Compared to podocin^wildtype/wildtype^ mice, podocin protein levels were decreased to 50% and 2.7% in podocin^Δexon5/wildtype^ and podocin^Δexon5/Δexon5^ mice, respectively. Nephrin protein levels were concomitantly changed albeit not to the same extent (Fig. 3d), whereas there was again no difference in *Nphs1* mRNA levels (Fig. 3e).

## STED microscopy and quantitative analysis reveal disruption of foot process morphology

**Fig. 4:**
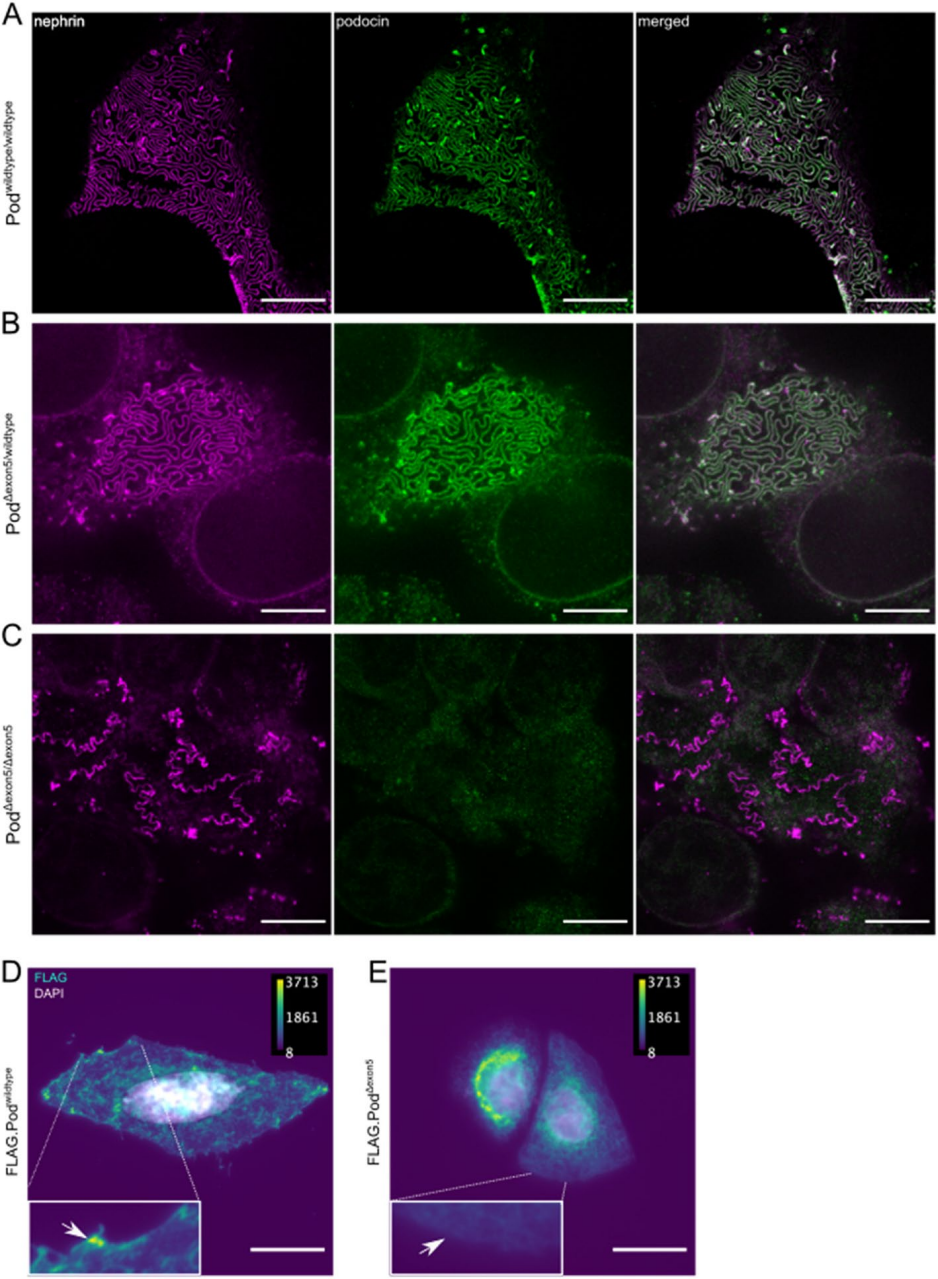
STED imaging reveals disrupted foot process morphology in podocin^Δexon5/Δexon5^ mice. **A**-**C** STED imaging following immunolabelling with antibodies against nephrin and podocin in samples of podocin^wildtype/wildtype^ (**A**), podocin^Δexon5/wildtype^ (**B**) and podocin^Δexon5/Δexon5^ (**C**) mice at P0. Scale bars correspond to 5 µm. (D)-(E) Conventional immunofluorescence microscopy of HeLa cells transiently transfected with FLAG-tagged podocin^wildtype^ (**D**) and podocin^Δexon5^ (**E**) confirms the retaining of podocin^Δexon5^ in the endoplasmic reticulum. Scale bars correspond to 20 µm

Applying STED microscopy to kidney sections of podocin^wildtype/wildtype^, podocin^Δexon5/wildtype^, and podocin^Δexon5/Δexon5^ mice on their day of birth after immunolabelling podocin and nephrin allowed the visualization of the podocyte foot process morphology and distribution of slit diaphragm-associated proteins (Fig. 4a-c) [19]. In Fig. 4a, the regular architecture with foot processes densely covering the capillary surface could be observed. While this staining pattern of podocin and nephrin appeared to be relatively unchanged between podocin^wildtype/wildtype^ and podocin^Δexon5/wildtype^ mice, ultrastructure in podocin^Δexon5/Δexon5^ mice was severely disrupted. In addition, a complete absence of podocin from the slit diaphragm complex became evident (Fig. 4c, middle panel). Importantly, we made sure that the podocin antibody used in this study was able to bind to the native as well as the denatured podocin^Δexon5^ variant by performing immunoprecipitation and immunoblot experiments (Suppl. Figure 2). The absence of podocin^Δexon5^ at the slit diaphragm could be explained by a previous study from our group showing that the human short isoform transiently expressed in HeLa cells is retained in the endoplasmic reticulum [15]. We have confirmed this finding in a similar experiment by transiently expressing the murine isoforms podocin^wildtype^ and podocin^Δexon5^ (Fig. 4d, e). Visualization of the two FLAG-tagged podocin variants clearly shows the enrichment of podocin^Δexon5^ to the region around the nucleus and its absence from the cell membrane (Fig. 4e). In contrast, there was a strong staining signal at the plasma membrane in cells expressing podocin^wildtype^ (Fig. 4d).

**Fig. 5:**
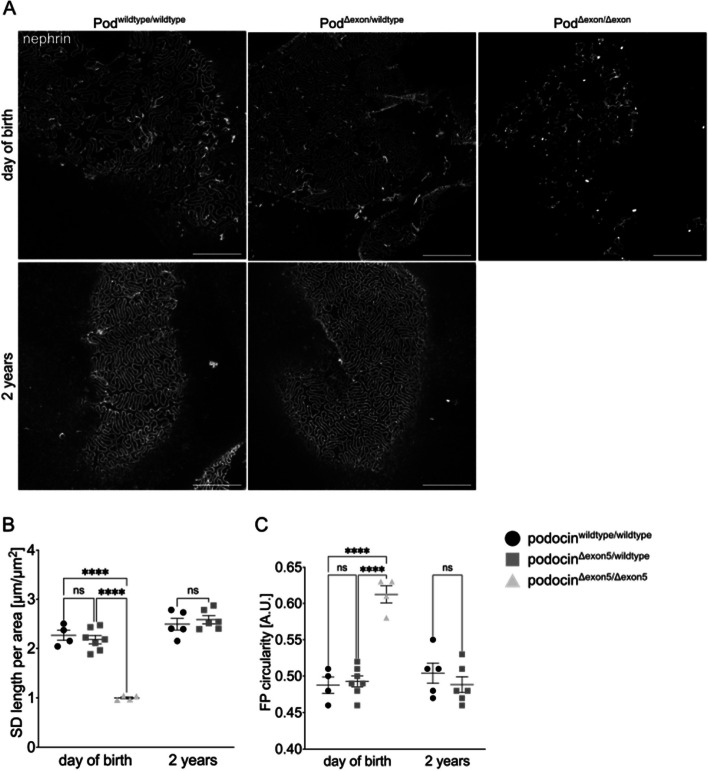
Comparison of podocyte morphology in young and aged podocin^wildtype/wildtype^, podocin^Δexon5/wildtype^ and podocin^Δexon5/Δexon5^ mice. **A** STED imaging following immunolabelling with an anti-nephrin antibody in young (day of birth) and aged (2 years) podocin^wildtype/wildtype^_,_podocin^Δexon5/wildtype^ and podocin^Δexon5/Δexon5^ mice. Scale bars correspond to 5 µm. **B**-**C** Quantification of SD length per area (**B**) and FP circularity (**C**) in young (day of birth) and aged (2 years) mice. Each dot/square represents one mouse. Tukey’s multiple comparison test was performed to determine statistical significance. **** *p* < 0.0001. ns = not significant. Data are presented as mean ± SEM

We have recently shown how STED microscopy and computational analyses of super-resolution images can be used to characterize morphological alterations of podocytes (see methods section and Suppl. Figure 3 for a detailed description of the parameters used) [20], which we further developed into a fully-automated, deep-learning-based pipeline to analyze podocyte morphology (Automatic Morphological Analysis of Podocytes = AMAP) in a streamlined and investigator-independent fashion [21]. In a follow-up study, we could demonstrate that this analysis tool can be used to detect subtle changes in podocyte morphology [22], which are thought to affect the physical forces contributing to podocyte loss [23]. We used this analysis workflow to demonstrate morphological differences between wildype mice and mice heterozygous for a frequent missense variant in *Nphs2* [22]. To quantify the effect of the above-mentioned decreased protein levels of podocin and nephrin on podocyte foot process morphology, we systematically analysed the slit diaphragm (SD) length per area and foot process (FP) circularity in podocin^wildtype/wildtype^, podocin^Δexon5/wildtype^ and podocin^Δexon5/Δexon5^ mice at the day of birth and podocin^wildtype/wildtype^ and podocin^Δexon5/wildtype^ mice at 2 years of age, using our recently developed AMAP tool [21]  (Fig. 5A-C). While STED imaging following nephrin immunolabelling did not reveal apparent differences between podocin^wildtype/wildtype^ and podocin^Δexon5/wildtype^ mice at either time point, there were significant changes in podocin^Δexon5/Δexon5^ mice as compared to their podocin^wildtype/wildtype^ and podocin^Δexon5/wildtype^ littermates (Fig. 5B-C). Automated quantification of SD length per area and FP circularity confirmed this, as there were significant differences in global SD abundance (SD length per area) and individual FP morphology (FP circularity) between podocin^Δexon5/Δexon5^ mice and the other genotypes in newborn pups. Between podocin^wildtype/wildtype^ and podocin^Δexon5/wildtype^ mice there was not even in aged mice a significant difference in foot process morphology.

## Discussion

Consistent with our previous findings in cell culture on human podocin lacking exon 5, we show that the murine podocin^Δexon5^ is expressed and is similarly mislocalized to the endoplasmic reticulum [15]. Contrary to the cell culture findings, podocin protein levels were massively decreased in podocin^Δexon5/Δexon5^ mice. This illustrates how efficiently mislocalized podocin is degraded in vivo. It was previously shown that podocin R138Q, a variant known to be retained in the ER, is mostly degraded via the proteasome rather than the lysosome and it is intriguing to assume a similar fate for podocin^Δexon5 ^[17]. Using STED microscopy, we could not detect any podocin^Δexon5^ in vivo at the slit diaphragm protein complex in podocyte foot processes of podocin^Δexon5/Δexon5^ mice. Despite the podocin^Δexon5^ mRNA being present at comparable amount to the wildtype mRNA, we could hardly detect the corresponding protein. Our characterization of the podocin^Δexon5^ mouse line illustrates the effect of the variant on the organism and the importance of podocin for the integrity of the glomerular filtration barrier: the absence of podocin from the slit diaphragm protein complex at the foot process severely compromised the normal foot process morphology and glomerular function, as became evident from the early death. This observation is yet another example of the inseparability of morphology and function in the mammalian glomerulus. The phenotype of podocin^Δexon5/Δexon5^ mice described in this study was very similar to that of podocin-deficient mice [6] and appears to be more severe than the *Nphs2* R140Q mouse [18], which leaves the physiological role of the human short isoform of podocin unclear. Based on the findings of this study, it can, however, be stated that the human short isoform is unlikely to have a significant rescue potential in cases in which the canonical form of podocin is altered by mutations in exon 5. Another striking observation was the inability of podocin^Δexon5/Δexon5^ mice to produce similar amounts of urine as compared to podocin^wildtype/wildtype^ and podocin^Δexon5/wildtype^ mice. As no sclerotic lesions were found, which potentially could have clogged the glomerular capillaries, thereby preventing filtration, our findings confirm the hypothesis to attribute podocin a central role in orchestrating the installation of a functioning filtration slit [6]. The observation also supports this central role: decreased podocin levels in podocin^Δexon5/wildtype^ mice are accompanied by reduced nephrin protein levels. Interestingly, decreased protein levels of these two central slit diaphragm proteins did not affect the podocyte ultrastructure, indicating that these lower levels can be compensated on a morphological level.

In summary, we did not find an indication for a physiological role of the short isoform in vivo in the human kidney, which is surprising given the evident mRNA expression in relevant quantities. The detrimental effect of podocin’s absence at the morphological and functional level in homozygous podocin^Δexon5/Δexon5^ mice not only underscores podocin’s role in maintaining the glomerular filtration barrier but also sheds light on its relevance in the formation of the regular slit diaphragm architecture. As to the function of the short isoform, it remains to be investigated whether it has a physiological extrarenal role, e.g., in the testes where it is also expressed on the RNA level.

## Methods

### Animal models

All mouse experiments were conducted in accordance with European, national and institutional guidelines and were approved by the State Office of North Rhine-Westphalia, Department of Nature, Environment and Consumer Protection (LANUV NRW, Germany; animal approval AZ 81–02.04.2018.A325 and AZ 84-02_04_2014_A372). All methods are reported in accordance with ARRIVE guidelines for the reporting of animal experiments. Mice were kept in the specific and pathogen free animal facility of the CECAD Research Center, University of Cologne, Germany, in individually ventilated cages (Greenline GM500m Tecniplast) at 22 °C and a humidity of 55% under 12 h light cycle with access to water and food ad libitum. Breeding and genotyping was performed according to standard procedures. Podocin^Δexon5^ mice were generated in our in vivo research facility (CECAD Research Center University of Cologne, Germany) as will be described below. All experiments were conducted in a pure C57BL6/NRj background and in male and female mice (combined analyses are presented). The age of the animals used for the respective experiments is indicated in the figures and/or the figure legends.

### Generation of guide RNAs and repair templates

The sgRNA used was generated by T7 RNA polymerase mediated in vitro transcription (ThermoFisher, A29377) and column purified (Qiagen, 217004). All protocols were conducted according to the manufacturer’s instructions. Guide RNA was stored at -80 °C. Custom single stranded donor oligonucleotides (ssODN) were ordered from IDT, resuspended in nuclease-free H_2_O to a final concentration of 10 µM and stored at -80 °C. sgRNA and ssODN sequences are listed in the Key Resources Table. The ssODN was originally designed to introduce a point mutation in order to generate an amino acid change at position p.234 (p.T234I). In the podocin ^Δexon5^ mouse line, the occurrence of non homologous end joining lead to the deletion of one splice site of exon 5 with subsequent in-frame skipping of the whole exon.

### Mouse transgenesis

Mouse transgenesis was performed as previously described [24]. In brief, embryos for microinjection were collected from the oviducts of superovulated donor females (100% C57BL6/NRj background, Janvier Labs (SE-ZYG-CNP)). Microinjections were carried out with the help of an Axio Observer.D1 microscope (Zeiss) and microinjector devices CellTram and FemtoJet with TransferMan NK2 micromanipulators (Eppendorf). A premixed solution containing the sgRNA (50 ng/µl), Cas9 mRNA (50 ng/µl; TriLink Biotechnologies), Cas9 protein (30 ng/µl; PNA Bio Inc) and the ssODNs(100 ng/µl; IDT) was injected into the male pronucleus with injection capillaries (BioMedical Instruments, BM 100F-10; type PI-1.6) [25]. One day after the microinjection, 2-cell stage embryos were transferred into the oviducts of pseudo-pregnant foster females.

### Genotyping

Mice were genotyped using DNA isolated from ear biopsies or tail tissue. Genomic DNA was amplified by PCR using REDTaq ReadyMix (Sigma Aldrich) and visualized via gel electrophoresis. Primers used were: 5’-ACTGACTGACTGATTCCCCA-3’ and 5’-GCCCGGCTCTATGCTATAAT-3’. Sanger Sequencing of the amplified fragment in podocin^Δexon5/Δexon5^ mice confirmed a 255 base pair deletion (genomic region 10,261–10,515 from NCBI Reference Sequence NC_000067.7).

### Urine analyses

Urine samples were analyzed using albumin ELISA (mouse albumin ELISA kit; Bethyl Labs, Montgomery, TX, USA) and a creatinine assay (Cayman Chemical, Ann Arbor, MI, USA). In order to obtain the urinary albumin creatinine ratio (ACR), albumin concentrations were divided by creatinine concentrations. For the coomassie staining 0.5 µl urine was diluted 1:20 with 1 × laemmli buffer with DTT and run on a 10% sodium dodecyl sulphate–polyacrylamide gel electrophoresis (SDS-PAGE). Subsequently, the gels were stained with a colloidal coomassie staining solution and scanned using a LI-COR Odyssey CLx scanner (LI-COR Biotechnology, Lincoln, Nebraska, USA).

### Histology and immunohistochemistry

Experimental mice were euthanized by decapitation (only newborn mice) or cervical dislocation. After median laparotomy, kidneys were removed and fixed in 4% neutral buffered formalin for 2–4 h at room temperature or overnight at 4 °C. Subsequently, dehydration and embedding in paraffin was performed. Kidney tissue was cut using a microtome (Leica Biosystems, Nussloch, Germany) and sections were transferred on glass slides. To assess glomerulosclerosis Periodic acid Schiff (PAS) staining was performed. Briefly, 1-µm-thin sections were deparaffinized in Xylene (VWR, Darmstadt, Germany) and subsequently rehydrated in a descending ethanol row. After incubation for 10 min each in 0.9% periodic acid (Carl Roth, Karlsruhe, Germany), Schiff reagent (Merck, Darmstadt, Germany) and Mayer’s hematoxylin (Merck, Darmstadt, Germany) the sections were dehydrated in an ascending ethanol row and Xylene and covered with Histomount (National Diagnostics, Atlanta, GA, USA).

For Wilms Tumor Protein (WT-1) staining, 2 µm thick kidney sections were deparaffinized in Xylene (VWR, Darmstadt, Germany) and subsequently rehydrated in a descending ethanol row. After antigen retrieval in a microwave for 10 min in a buffer containing 10 mM Trizma base (Sigma Aldrich) and 10 mM Ethylenediaminetetraacetic acid disodium salt dehydrate (EDTA, Sigma Aldrich) at pH 9.0, sections were blocked in phosphate buffered saline (PBS; 137 mM NaCl, 2.7 mM KCl, 10 mM Na_2_HPO_4_, 1.8 mM KH_2_PO_4_). Subsequently, sections were incubated with 3% H_2_O_2_ for 15 min, then washed in PBS and blocked with rodent block M (Linaris). After washing with PBS, sections were incubated with a rabbit monoclonal antibody against mouse WT-1 (Abcam, Cat# ab89901, RRID:AB_2043201, 1:500) over night at 4 °C. After washing with PBS, sections were incubated with horseradish peroxidase polymer for 30 min at room temperature. After washing with PBS, sections were incubated in DAB (3,3’Diaminobenzidine) solution for 10 min at 37 °C and subsequently washed in tap water followed by an incubation with hematoxylin for 10 s. After another washing step in tap water, sections were dehydrated in an ascending ethanol row before mounting with Histomount (National Diagnostics, Atlanta, GA, USA). A Leica SCN400 slidescanner (Leica Biosystems, Nussloch, Germany) was used to acquire images that were further processed with ImageJ/Fiji software version 1.52i (NIH, Bethesda, MD, USA).

### RNA purification and quantitative PCR

Experimental mice were euthanized by decapitation. RNA from kidney cortex of podocin^wildtype/wildtype^, podocin^Δexon5/wildtype^ and podocin ^Δexon5/ Δexon5^ mice at P 0 (= day of birth) was isolated using the Zymo Research Direct-zol RNA miniprep kit (Zymo Research Europe, Freiburg, Germany, catalogue # R2052) according to the manufacturor’s instructions. Quality of the RNA was checked measuring absorption in a nanodrop (ThermoFisher Scientific). 500 ng RNA was used for reverse transcription using the High-Capacity cDNA Reverse Transcription Kit (ThermoFisher Scientific, catalogue # 4,368,814) accoring to the manufacturor’s instructions. Quantitative PCR was performed using SYBR Green and Taqman master mixes. 25 ng cDNA and primers for murine podocin and murine. QuantStudio 12 K Flex Real Time PCR System v1.2.2 (Thermo Fisher Scientific) was used for data analysis. Primers used were: 5'-ATTACTCTTTCATACTCTTGCACAAC-3’ and 5'-CATCAAGCCCTCTGGATTAGG-3' (murine *Nphs2*, both isoforms); 5'-CTTTCCATGAGGTTGCCTTAGA-3' and 5'-GGCAGCCTCACATCCTTAAT-3' (murine *Nphs2*, only Δexon5 isoform); 5’-CAGGGTCGGAGGAGGATCGAATC-3’ and 5’- GAAGCTCCACGGTTAGCACAGCAG-3’ (murine *Nphs1*). For murine *Hprt* a predesigned qPCR assay from IDT (Integrated DNA Technologies, Leuven, Belgium) was used.

### Sample preparation for LC–MS

Experimental mice were euthanized by decapitation. One half of kidney per mouse of podocin^wildtype/wildtype^, podocin^Δexon5/wildtype^ and podocin ^Δexon5/ Δexon5^ mice at P 0 (= day of birth) was processed for mass spectrometry. Samples were homogenized in 550 µl urea buffer 8 M containing 50 mM ammonium bicarbonate, placed in a bioruptor for 10 min and centrifuged for 60 min at 20.000 × g and 4 °C. 10 µl supernatant per sample was used the protein concentration in each sample. For reduction and alkylation, respectively, the remaining supernatant was incubated in 10 mM DTT and 50 mM CAA for 60 min at room temperature each. After this, 50 µg protein per sample was digested with 1 µg Trypsin at room temperature overnight. After acidic elution of the pepites, a stage-tip clean-up protocol was performed as previously described [26].

### Liquid chromatography-tandem mass spectrometry

A targeted assay was designed to quantify specific nephrin and podocin peptides within the kidney cortex samples (nephrin peptides: ELVLIIGPPDNLAK, SGSTFSR; podocin peptides: ARPDAGAER (amino acids 37–45), MAAEILSGTPAAVQLR (amino acids 309–324), VALDAVTCIWGIK (amino acids 249–261)). Samples were measured on a Q-Exactive HF-X mass spectrometer coupled to an Easy-nLC 1200 (both Thermo Scientific). Peptides were separated on a self-packed C18 column (50 cm length, 75 µm inner diameter) on the following gradient running 0.1% formic acid (buffer A) against 80% acetonitrile with 0.1% formic acid (buffer B) at a constant flow of 250 nl/min: initial 4% B, up to 29% B in 42 min, up to 55% B in 8 min, up to 95% B in 2 min, followed by column washing and recalibration.The mass spectrometer was operated in PRM mode with a resolution of 30,000, an AGC target of 2E5 and 200 ms maximum injection time. Isolation of peptides of interest was done in a 1 Th window with a 0.2 Th offset and fragmentation of them was performed at 35 normalized collision energy.Target peptides were afterwards analyzed in Skyline against a Prosit-simulated library [27] and only peptides with the highest dotp were used to compare peptide abundances (for nephrin: ELVLIIGPPDNLAK; for podocin: MAAEILSGTPAAVQLR).

### Cell culture

HeLa and HEK293T cells were cultured in DMEM supplemented with 10% fetal bovine serum under standard conditions (5% CO_2_, 37 °C). For transfection experiments, cells were grown to 60–80% confluency and transfected using the calcium phosphate method.

### Cloning, plasmids and transfection

For the generation of the podocin^Δexon5^ plasmid, podocin cDNA of a podocin ^Δexon5/ Δexon5^ mouse was cloned using the primers 5-taa ata acg cgt ATG gac agc agg gcg cgg agc-3 and 5´-cgcggggcggccgccttactataacataggagagtc-3´ and swapped into a modified pcDNA6 vector containing a FLAG or V5 tag, respectively. FLAG-tagged and V5-tagged podocin^wildtype^ and podocin ^Δexon5^ pcDNA6 constructs were used to transfect HeLa and HEK293T cells. Primers 5’- cgcggg acgcgt TGTCTGGACACCTATCACAAG-3’ and 5’- cgcggg gcggccgcc cta AGTTCTCTCCACTTTGATGCC-3’ were used to amplify cDNA of podocin^wildtype/wildtype^ and podocin^Δexon5/ Δexon5^ mice. PCR products were subsequently run on an agarose gel or used for Sanger sequencing.

### Immunoprecipitation

HEK293T cells were incubated for 24 h after transfection. After washing with cold PBS, cells were lysed in IP-buffer (1% Triton X-100; 20 mM Tris pH 7.5; 25 mM NaCl; 50 mM NaF; 15 mM Na_4_P_2_O_7_; 1 mM EDTA; 0.25 mM PMSF; 5 mM Na_3_VO_4_) at 4 °C for 15 min and subsequently centrifuged (18.000 rom, 4 °C, 15 min). Equal amounts of supernatant was incubated with 1 µg of rabbit anti-podocin primary antibody (Sigma-Aldrich, Cat# P0372, RRID:AB_261982) coupled to protein G-beads (GE healthcare) for 1 h at 4 °C. After washing the beads 3 times with IP buffer, lysates and precipitates were boiled in 2 × Laemmli buffer. Lysates and immunoprecipitated samples were subjected were subjected to western blotting.

### Immunoblotting

Cell lysates and precipitates of HEK293T cells were run on 10% sodium dodecyl sulphate–polyacrylamide gel electrophoresis (SDS-PAGE) and transferred on PVDF membranes. Membranes were blocked in 5% BSA in PBS and then incubated for 1 h at room temperature with a rabbit anti-podocin primary antibody (Sigma-Aldrich, Cat# P0372, RRID:AB_261982, 1:1000). Incubation with a goat anti-rabbit horse radish peroxidase antibody (Jackson ImmunoResearch Labs Cat# 111–035-008, RRID:AB_2337937, 1:15.000) for 1 h at room temperature and subsequent chemoluminescence-based detection using a Fusion Solo imager (Vilber Deutschland, Eberhardzell, Germany) allowed visualization of the proteins.

### Conventional immunofluorescence microscopy of HeLa cells

HeLa cells transiently expressing FLAG-tagged podocin^wildtype^ and podocin^Δexon5^ were seeded on coverylips at 37 °C overnight. On the following day, the cells were fixed with 4% paraformaldehyde (PFA) for 8 min at room temperature after washing with phosphate buffered saline (PBS). Cells were blocked with 5% normal donkey serum in PBST for 30 min at room temperature and incubated with a mouse monoclonal anti-FLAG antibody (Sigma-Aldrich Cat# F3165, RRID:AB_259529, 1:1000) at 4 °C overnight and a donkey anti-mouse cy3 secondary antibody (Jackson ImmunoResearch Labs Cat# 715–165-150, RRID:AB_2340813, 1:500) for 1 h at room temperature. Samples were mounted in ProLong Diamond antifade with DAPI (Thermo Fisher Scientific). An Axio Observer microscope with ZEN software version 2.6 (both Carl Zeiss, Germany) was used to acquire images that were further processed with ImageJ/Fiji software version 1.52i (NIH, Bethesda, MD, USA) [28].

### Optical clearing of kidney sections

After formalin fixation (see “Histology and immunhistochemistry”), pieces of kidney were incubated at 4 °C in hydrogel solution (HS) (4% v/v acrylamide, 0.25% w/v VA-044 initiator, PBS 1X) over night. The gel was polymerized at 37 °C for 3 h, and the presence of oxygen was minimized by filling tubes all the way to the top with HS. Samples were removed from the HS and immersed in clearing solution (CS) (200 mM boric acid, 4% SDS, pH 8.5) at 50 °C for 6 h. After that, kidney pieces were cut into 0.3 mm thick slices using a Vibratome. Slices were then incubated at 50 °C overnight. Prior to immunolabelling, samples were washed in PBST (0.1% Triton-X in 1X PBS) for 10 min.

### Immunolabelling of kidney sections

For all steps PBST (PBS 1X with 0.1% v/v Triton-X) was used as diluent. Samples were incubated in primary antibody for 24 h at 37 °C, and were then washed in PBST for 10 min at 37 °C followed by secondary antibody incubation for 24 h at 37 °C and washed for 10 min at 37 °C prior to mounting. To stain for podocin a rabbit anti-podocin primary antibody (Sigma-Aldrich, Cat# P0372, RRID:AB_261982, 1:100) and a donkey anti-rabbit Atto-594 (mouse samples, see below, 1:50) or Alexa-555 (human patient samples, Thermo Fisher, A-31572, 1:100) secondary antibody was used. To stain for nephrin a sheep anti-nephrin primary antibody (R&D Systems AF4269 1:100) and a donkey anti-sheep Abberior STAR 635P (mouse samples, see below, 1:50) or donkey anti-goat Alexa-405 (human samples, Abcam, ab175664, 1:100) secondary antibody was used. Secondary antibodies used for mouse samples (STED microscopy) were conjugated in-house as follows. A fluorophore (Atto-594 NHS ester, Sigma Aldrich Cat# 08741 or Abberior STAR 635P NHS ester, Sigma Aldrich Cat# 07679) was conjugated to a donkey anti-sheep IgG (Thermo Fisher Scientific Cat# A16050, RRID:AB_2534723) or a donkey anti-rabbit IgG (Thermo Fisher Scientific Cat# A16037, RRID:AB_2534711) antibody. To this end, 1 M NaHCO_3_ was added to the antibody vials at a dilution of 1:10. The fluorophores were dissolved in dimethylsulfoxide at a concentration of 10 mg/ml. The fluorophores were added to the antibody solutions at a 20-fold molar excess of fluorophore to antibody followed by incubation on a shaker at room temperature for 1 h. Excess fluorophores were removed by using a centrifugal filter (Amicon Ultra 0.5 centrifugal filter 30 MW cutoff, Sigma-Aldrich Cat# UFC5030) and spinning down at 14,000 g for 10 min, then filling up with PBS containing 0.1% sodium azide and spinning down at 14,000 g for 10 min again. PBS containing 0.1% sodium azide was added for a final antibody concentration of 1 mg/ml. The fluorophore-conjugated antibodies were aliquoted and stored at -20 °C until use.

### Mounting and imaging of kidney sections

Samples were immersed in 80% (w/w) fructose with 0.5% (v/v) 1-Thioglycerol at 37 °C for 1 h prior to imaging. Samples were then mounted in a glass bottom dish (MatTek P35G-1.5–14-C) and imaged using a Leica SP8 3X STED system (Leica Microsystems, Germany).

### Quantification of morphometric parameters

For quantification of morphometric parameters, nephrin fluorescence images were further analyzed using our recently established method of Automatic Morphological Analysis of Podocytes (AMAP) [21].

In brief, for the SD length the capillary surface captured in an image was automatically assigned and the length of the SD, represented by the nephrin signal, was quantified automatically together with the area of the assigned region. For circularity (circularity = 4 π (area/perimeter^2)), individual foot processes were automatically segmented using instance segmentation. FP circularity is a dimensionless unit describing the shape of a geometric body. A value of 1 indicates a flawless circle and as the value approaches 0, it indicates an elongated polygon. The results were then exported as CSV file for further processing with Microsoft Excel and Graphpad Prism. A mean number of 1592 and a minimum of 648 foot processes were examined per animal. At least 4 glomeruli per animal were used for the quantification of the indicated morphometric parameters.

### Supplementary Information

**Additional file 1: Supplemental Fig. 1. **Amino acid alignment of human podocin canonical form, human podocin short isoform, murine podocin canonical form, exon 5 of human podocin and exon 5 of murine podocin. Amino acids are color coded for better visualization of alignment. The alignment was done using the T-Coffee webserver [23] and formatted using Jalview [24].** Supplemental Fig. 2. **The podocin antibody used in this study is able to bind to the denatured and the native podocin^Δexon5^ protein. Lysates of HEK293T cell transiently expressing podocin^wildtype^ or podocin^Δexon5^ were subjected to immunoprecipitation with an anti-podocin antibody and subsequent immunoblotting. The full-length blot is presented in suppl. fig. 5.** Supplemental Fig. 3. **Schematic display of the two morphological parameters used. An original image, labelled with an anti-nephrin antibody, is used to quantify the (1) SD length (yellow lines), represented by the length of the nephrin signal, within a region of interest (cyan line) and (2) the FP circularity, which is a dimensionless-less value expressing how circular a geometric body is (circularity = 4 * π (area/perimeter²)). A perfect circle has a value of 1, an elongated polygon approximates 0.** Supplemental Fig. 4. **Full-length gel of panel D of figure 1. Numbers refer to individual mice. Samples 3 and 4 are depicted in fig. 1.** Supplemental Fig. 5. **Full-length gel of panel C of figure 2.** Supplemental Fig. 5. **Full-length blot of suppl. fig. 2.

## Data Availability

The mass spectrometry proteomics data have been deposited to the ProteomeXchange Consortium via the PRIDE partner repository with the dataset identifier PXD041055. Source data for this study were derived from the GTEx Portal on 11/15/2022. Data Source: GTEx Analysis Release V8 (dbGaP accession number phs000424.v8.p2).
