# Supplementary material for: In vivo characterization of a podocyte-expressed short podocin isoform
Source: BMC Nephrol. 2023 Dec 19;24:378. doi: 10.1186/s12882-023-03420-x (PMC10731740; doi:10.1186/s12882-023-03420-x)
Supplement: Supplementary file 1 — Additional file 1: Supplemental Fig. 1. Amino acid alignment of human podocin canonical form, human podocin short isoform, murine podocin canonical form, exon 5 of human podocin and exon 5 of murine podocin. Amino acids are color coded for better visualization of alignment. The alignment was done using the T-Coffee webserver [23] and formatted using Jalview [24]. Supplemental Fig. 2. The podocin antibody used in this study is able to bind to the denatured and the native podocinΔexon5 protein. Lysates of HEK293T cell transiently expressing podocinwildtype or podocinΔexon5 were subjected to immunoprecipitation with an anti-podocin antibody and subsequent immunoblotting. The full-length blot is presented in suppl. fig. 5. Supplemental Fig. 3. Schematic display of the two morphological parameters used. An original image, labelled with an anti-nephrin antibody, is used to quantify the (1) SD length (yellow lines), represented by the length of the nephrin signal, within a region of interest (cyan line) and (2) the FP circularity, which is a dimensionless-less value expressing how circular a geometric body is (circularity = 4 * π (area/perimeter²)). A perfect circle has a value of 1, an elongated polygon approximates 0. Supplemental Fig. 4. Full-length gel of panel D of figure 1. Numbers refer to individual mice. Samples 3 and 4 are depicted in fig. 1. Supplemental Fig. 5. Full-length gel of panel C of figure 2. Supplemental Fig. 5. Full-length blot of suppl. fig. 2. [file 12882_2023_3420_MOESM1_ESM.docx]

**SUPPLEMENTAL MATERIAL**


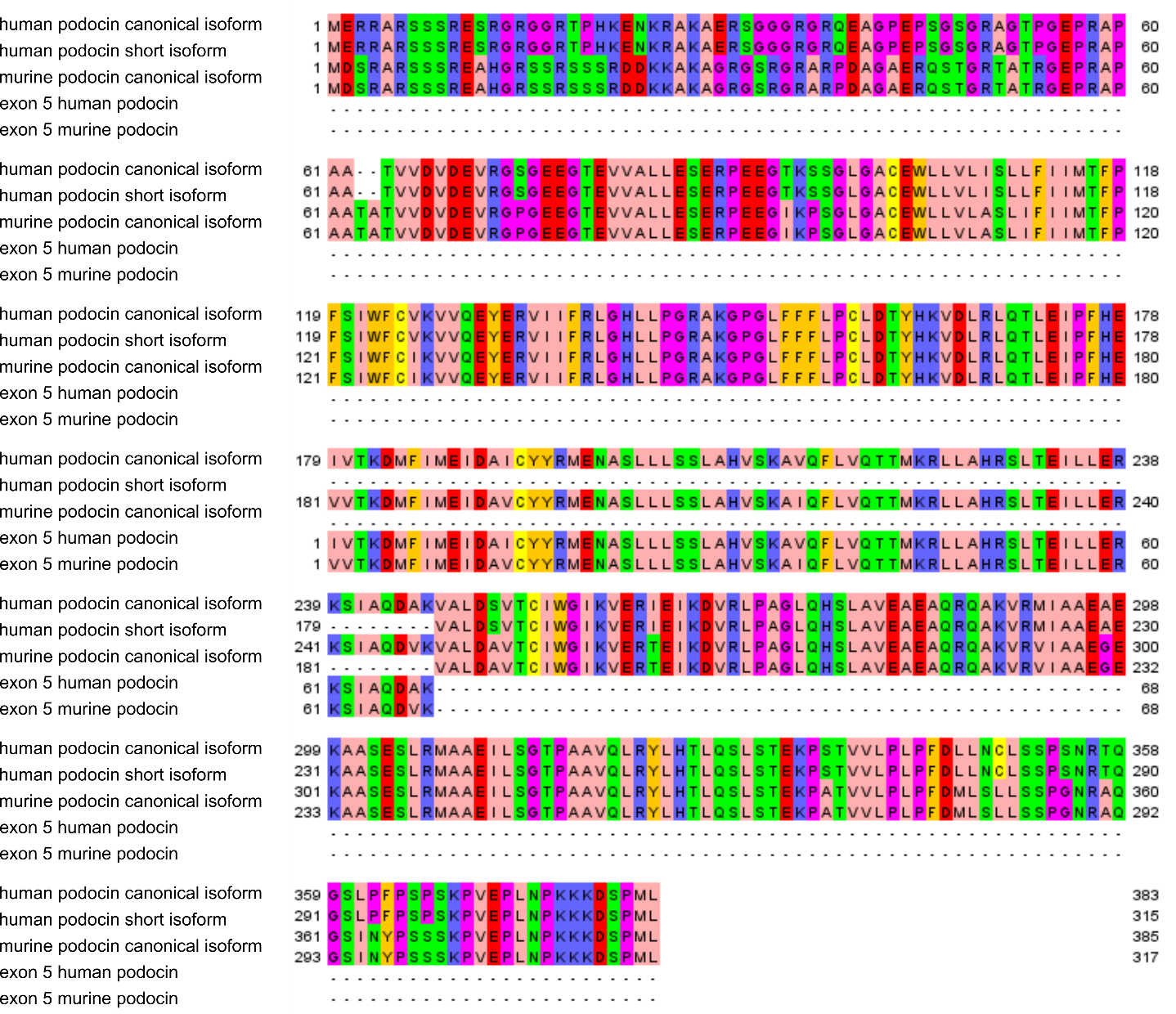


**Supplemental Fig. 1:** Amino acid alignment of human podocin canonical form, human podocin short isoform, murine podocin canonical form, exon 5 of human podocin and exon 5 of murine podocin. Amino acids are color coded for better visualization of alignment. The alignment was done using the T-Coffee webserver ^23^ and formatted using Jalview ^24^.


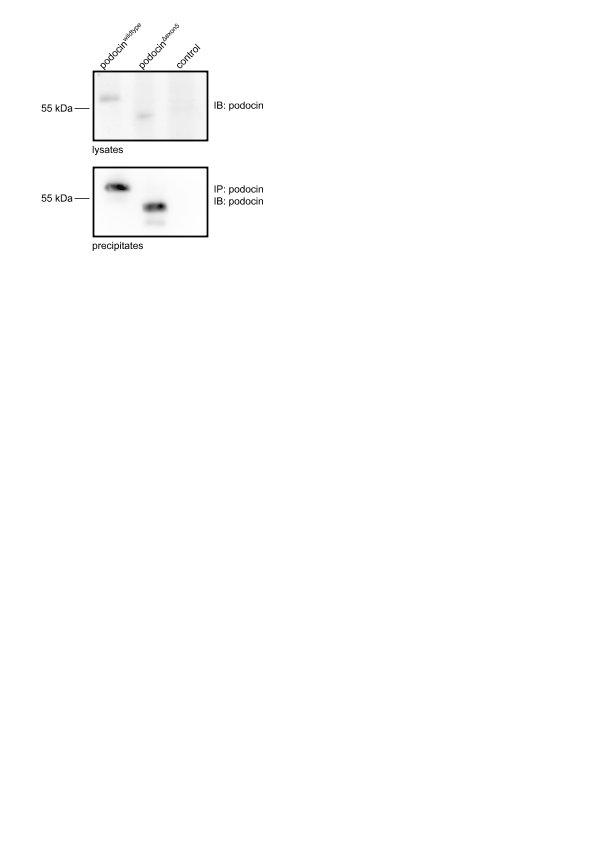


**Supplemental Fig. 2:** The podocin antibody used in this study is able to bind to the denatured and the native podocin^Δexon5^ protein. Lysates of HEK293T cell transiently expressing podocin^wildtype^ or podocin^Δexon5^ were subjected to immunoprecipitation with an anti-podocin antibody and subsequent immunoblotting. The full-length blot is presented in suppl. fig. 5.


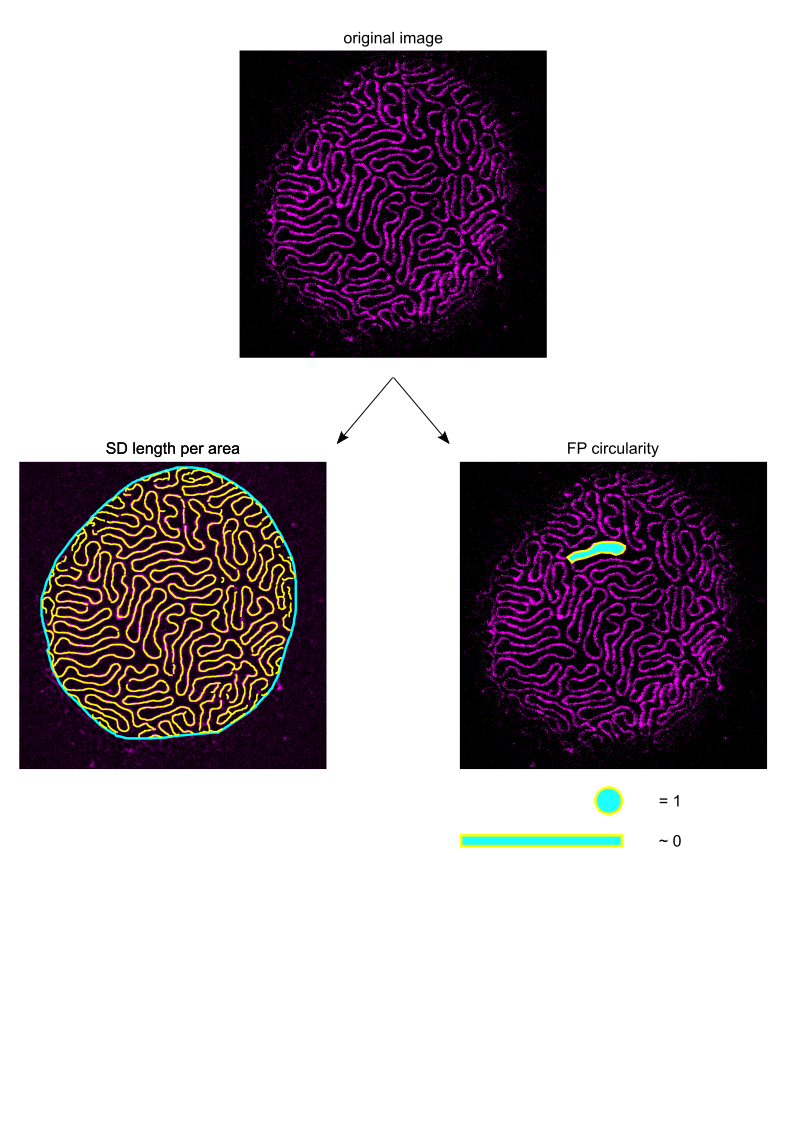
 **Supplemental Fig. 3:** Schematic display of the two morphological parameters used. An original image, labelled with an anti-nephrin antibody, is used to quantify the (1) SD length (yellow lines), represented by the length of the nephrin signal, within a region of interest (cyan line) and (2) the FP circularity, which is a dimensionless-less value expressing how circular a geometric body is (circularity = 4 * π (area/perimeter²)). A perfect circle has a value of 1, an elongated polygon approximates 0.


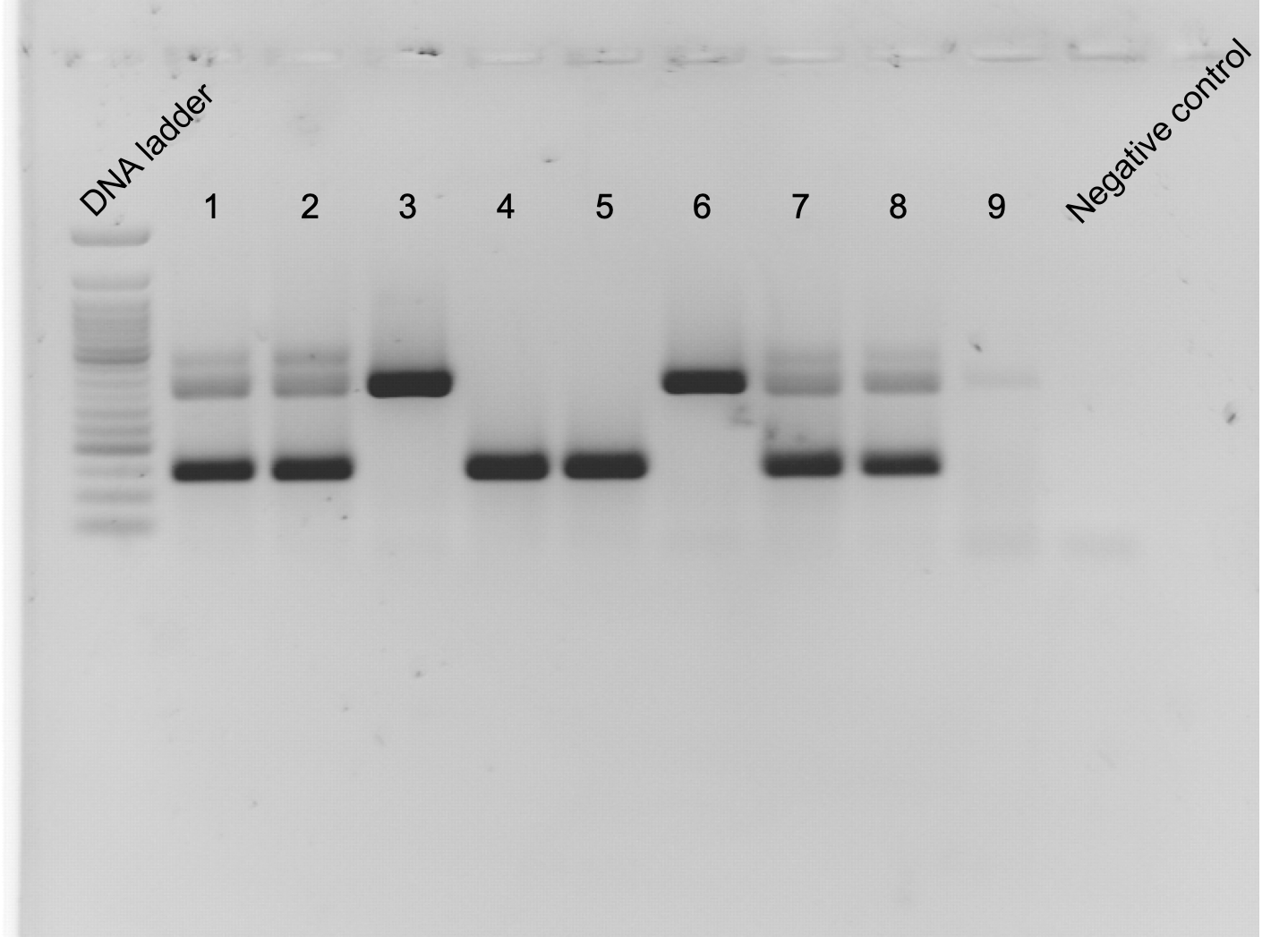


**Supplemental Fig. 4:** Full-length gel of panel D of figure 1. Numbers refer to individual mice. Samples 3 and 4 are depicted in fig. 1.


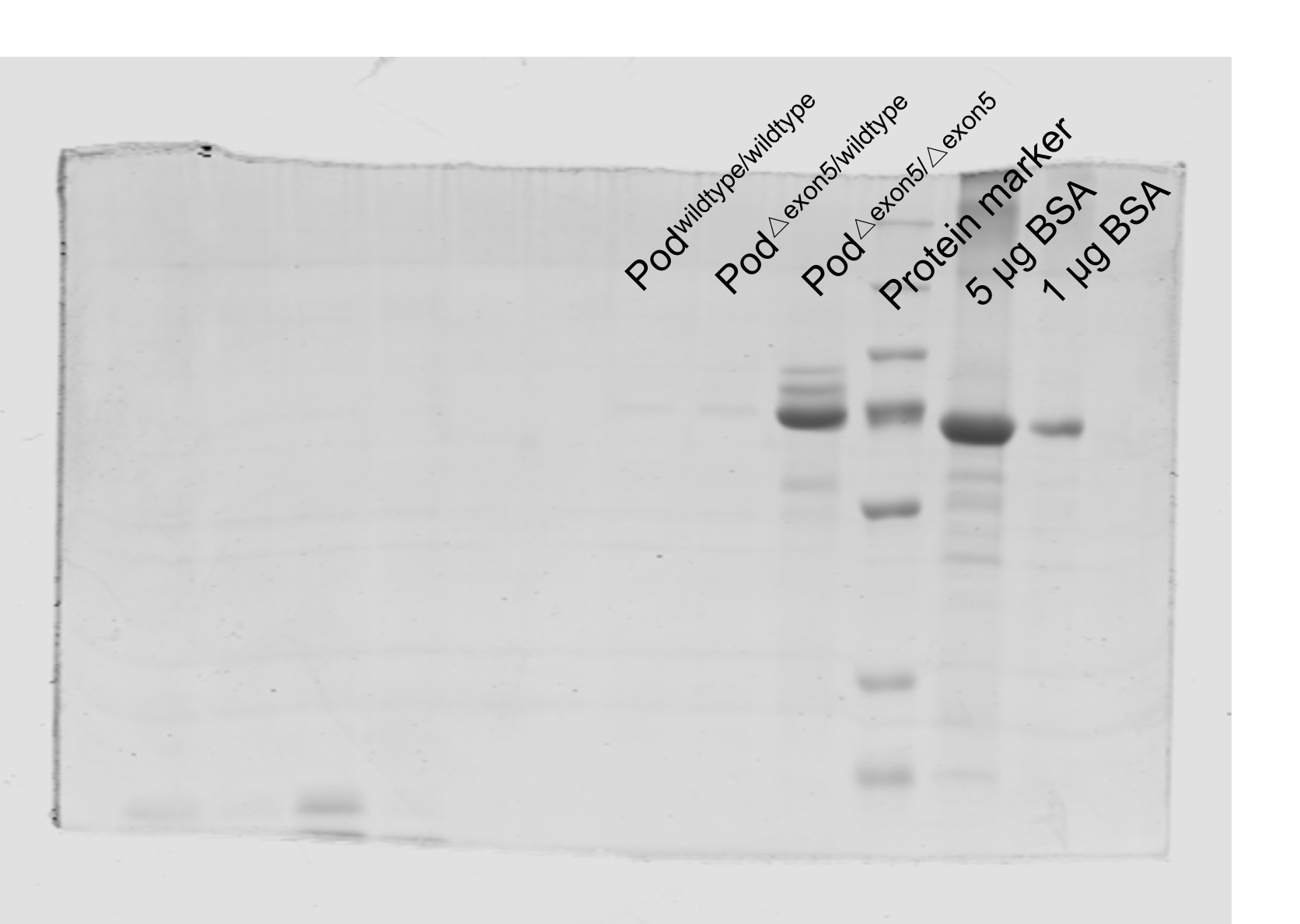


**Supplemental Fig. 5:** Full-length gel of panel C of figure 2.


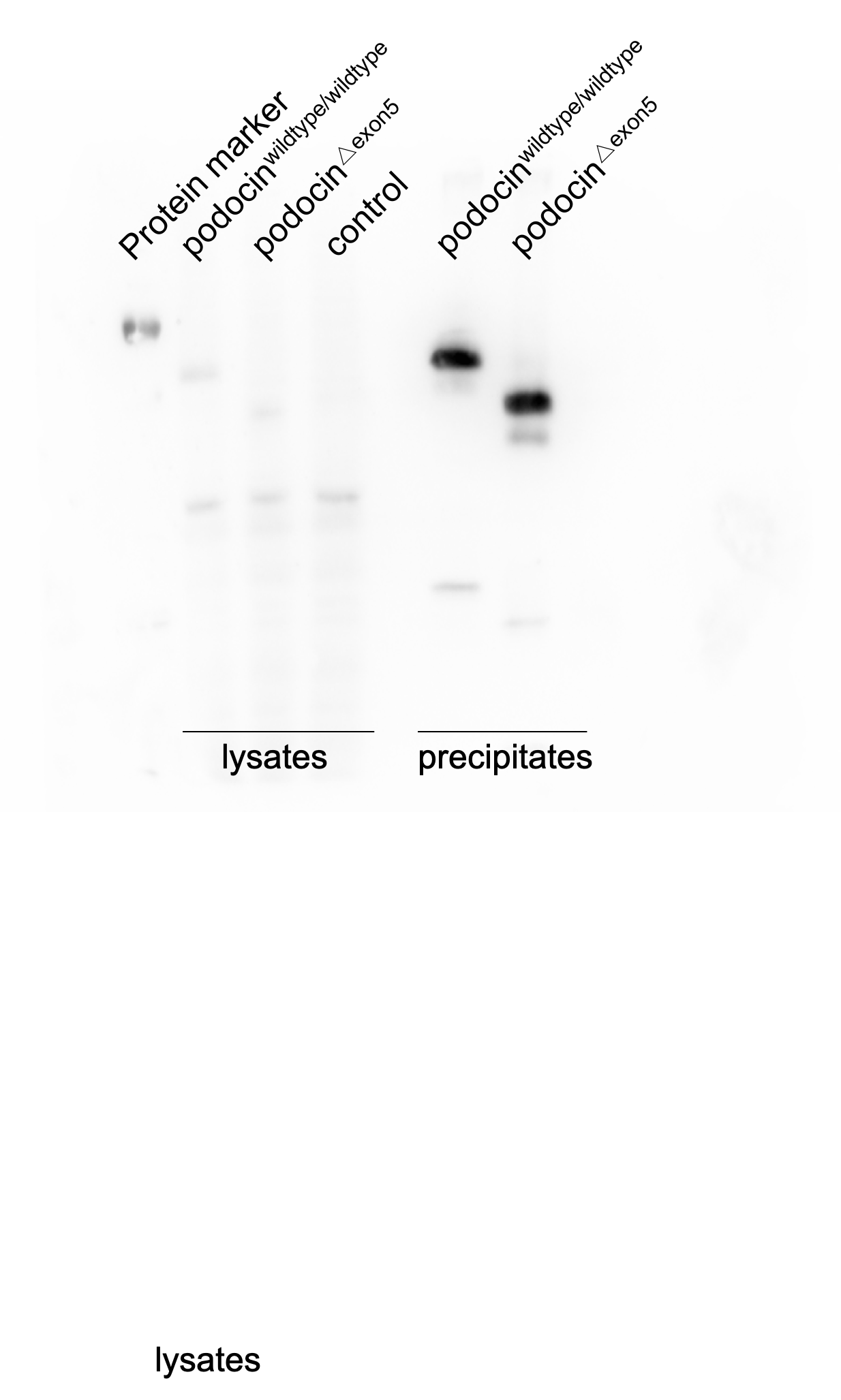


**Supplemental Fig. 5:** Full-length blot of suppl. fig. 2
